# A Remarkable Case of Hydrocephalus

**Published:** 1801-07

**Authors:** 


### *A remarkable Case of Hydrocephalus. By Dr.* Schmitt.

The accumulation of watery humours in the brain, and its cavities, is by no means an uncommon affection, though it has been more frequently obſerved in the preſent times than formerly; a circumſtance which ſeems to ariſe from its being then miſ-underſtood for other diſeaſes to which infants are expoſed. It is known that it generally occurs in the tender infantile age, and that the mortality of children in the earlier periods of life, derives a frequent origin from this ſource; but caſes of its being obſerved in more advanced ages are rare, and deſerve to be recorded in the annals of medicine. The Hydrocephalus which I am going to deſcribe, is one of the moſt remarkable inſtances inftances in its kind that has ever been mentioned. The patient is twenty-four years of age, and a male. The fkull is of a monftrous fize, every where offified, and its weight very extraordinary. Its circumference amounts to 25 inches, and the diameter, from the radix nafi to the protuberantia occipitalis, is about 17 inches. The body itfelf is proportionably fmall, like that of a boy, as its meafure is only, with the height of the head, 3 feet 5 inches, and, without the head, 2 feet 9 inches. The extremities are very thin and fmall, quite paralytic and contracted, the upper extremities particularly; the hands are in a fingular manner contorted, and lie clofe to the inner fide of the fore arm, which has been occafioned by the frequent convulfive fits that come on every day, generally lafting for about a quarter of an hour. The thorax is confiderably attenuated towards its bafis, but the back bone bent inwardly, and excavated, as it were, with a furrow; the os facrum is very ftrong, being fhaped like a vault. The head, however, remains the moft remarkable piece of the whole. All parts of this enormous fkull are tolerably proportioned, except the maxilla inferior, which being fmall, bears no proportion at all with the whole; on which account, the teeth of both jaws are not fituated one upon another, but thofe of the upper jaw are prominent over the teeth of the maxilla inferior: A natural confequence of this difproportion is, that the teeth become unfit for maftication, and the poor creature is not able to bite any thing; all his food muft be fluid, pappy, or minced, fo that he needs only to fwallow it. His digeftion is good, but his alvine excretion never proceeds by itfelf, as he is obliged to take a purgative, tea of fenna and manna, which they give him every fix or eight days, and which carries away a great quantity of fæces: The urine goes off involuntarily. His fpeech is very imperfect, and confifts of fome half articulated founds, which are only underftood by the people that are daily about him. There is, however, no perceptible fault in the formation of the tongue. The eyes are large, unfteady, and diftorted, and the pupils very much dilated; his mouth is extremely large, and awry. His look and phyfiognomy exprefs the higheft degree of weaknefs of intellect; it is the mien of a fheep or of a fool. It is very difficult to excite his attention, or to fix his continually upwards fquinting eyes upon any fubject; he is without any paffion; one may however make him laugh, but his laughing is rather a grin, which is extremely difgufting. The regio pubis is covered with long and thin hairs, and erections are fometimes obferved, but without any ejaculatio feminis. He knows nobody but the perfons that are always about him, viz. his father, a labourer; his mother, ther; and ſiſter, a perſon twenty-ſix years of age, tolerably well-made and healthy. His mother is already advanced in years, but ſtout and healthy; ſhe had eleven children, of which only three are ſurviving, a daughter, a ſon, and the Hydrocephalicus. According to the mother's account, he ſuffered much of head-ache the firſt years of his exiſtence, which ceaſing by degrees left him in this ſtate of ſenſeleſſneſs. It is ſurpriſing how fond his mother is of him, what pains ſhe takes to nurſe him, as the poor creature, unfit for any voluntary motion, wholly depends on others for all his neceſſities. Another inſtance of an Hydrocephalus, twenty-nine years of age, is deſcribed by Dr. Michaelis, in the Medical Communications, Vol. I. 1784. It is ſimilar to the above, though his mental faculties were leſs deranged.

